# Identification of Biochemical Determinants for Diagnosis and Prediction of Severity in 5q Spinal Muscular Atrophy Using ^1^H-Nuclear Magnetic Resonance Metabolic Profiling in Patient-Derived Biofluids

**DOI:** 10.3390/ijms252212123

**Published:** 2024-11-12

**Authors:** Afshin Saffari, Moritz Niesert, Claire Cannet, Astrid Blaschek, Andreas Hahn, Jessika Johannsen, Musa Kockaya, Heike Kölbel, Georg F. Hoffmann, Peter Claus, Stefan Kölker, Wolfgang Müller-Felber, Andreas Roos, Ulrike Schara-Schmidt, Friedrich K. Trefz, Katharina Vill, Wolfgang Wick, Markus Weiler, Jürgen G. Okun, Andreas Ziegler

**Affiliations:** 1 Division of Child Neurology and Metabolic Medicine, Department of Pediatrics I, Center for Pediatrics and Adolescent Medicine, Medical Faculty Heidelberg, University Hospital Heidelberg, Heidelberg University, 69120 Heidelberg, Germanystefan.koelker@med.uni-heidelberg.de (S.K.); friedrich.trefz@metabolic-consulting.de (F.K.T.); juergen.okun@med.uni-heidelberg.de (J.G.O.); 2 Department of Pediatrics I, Center for Pediatrics and Adolescent Medicine, Medical Faculty Heidelberg, University Hospital Heidelberg, Heidelberg University, 69120 Heidelberg, Germanygeorg.hoffmann@med.uni-heidelberg.de (G.F.H.); 3 Bruker BioSpin GmbH & Co. KG, 76275 Ettlingen, Germany; 4 Department of Pediatrics, Division of Pediatric MUC iSPZ Hauner—Munich University Center for Children with Medical and Developmental Complexity, Dr. von Hauner Children’s Hospital, LMU University Hospital, 80337 Munich, Germany; astrid.blaschek@med.uni-muenchen.de (A.B.); wolfgang.mueller-felber@med.uni-muenchen.de (W.M.-F.); katharina.vill@med.uni-muenchen.de (K.V.); 5 Department of Child Neurology, University Hospital Giessen, 35392 Giessen, Germany; andreas.hahn@paediat.med.uni-giessen.de; 6 Department of Pediatrics, Neuropediatrics, University Medical Center Hamburg-Eppendorf, 20246 Hamburg, Germany; j.johannsen@uke.de; 7 Buchener Straße 12, 68259 Mannheim, Germany; dr.kockaya@web.de; 8 Department of Neuropediatrics, Developmental Neurology and Social Pediatrics, Centre for Neuromuscular Disorders in Children, Children’s University Clinic Essen, University of Duisburg-Essen, 45147 Essen, Germany; heike.koelbel@uk-essen.de (H.K.); andreas.roos@uk-essen.de (A.R.); ulrike.schara-schmidt@uk-essen.de (U.S.-S.); 9 SMATHERIA gGmbH—Non-Profit Biomedical Research Institute, 30625 Hannover, Germany; 10 Institute of Functional and Applied Anatomy, Hannover Medical School, 30625 Hannover, Germany; 11 Center for Systems Neuroscience, 30625 Hannover, Germany; 12 Department of Neurology, Medical Faculty Heidelberg, University Hospital Heidelberg, Heidelberg University, 69120 Heidelberg, Germany; wolfgang.wick@med.uni-heidelberg.de (W.W.); markus.weiler@med.uni-heidelberg.de (M.W.); 13 Center for Pediatrics and Adolescent Medicine, Pediatric Clinical-Pharmacological Trial Unit (paedKliPS), Medical Faculty Heidelberg, University Hospital Heidelberg, Heidelberg University, 69120 Heidelberg, Germany

**Keywords:** spinal muscular atrophy, ^1^H-NMR spectroscopy, metabolic profiling, metabolomics

## Abstract

This study explores the potential of ^1^H-NMR spectroscopy-based metabolic profiling in various biofluids as a diagnostic and predictive modality to assess disease severity in individuals with 5q spinal muscular atrophy. A total of 213 biosamples (urine, plasma, and CSF) from 153 treatment-naïve patients with SMA across five German centers were analyzed using ^1^H-NMR spectroscopy. Prediction models were developed using machine learning algorithms which enabled the patients with SMA to be grouped according to disease severity. A quantitative enrichment analysis was employed to identify metabolic pathways associated with disease progression. The results demonstrate high sensitivity (84–91%) and specificity (91–94%) in distinguishing treatment-naïve patients with SMA from controls across all biofluids. The urinary and plasma profiles differentiated between early-onset (type I) and later-onset (type II/III) SMA with over 80% accuracy. Key metabolic differences involved alterations in energy and amino acid metabolism. This study suggests that ^1^H-NMR spectroscopy based metabolic profiling may be a promising, non-invasive tool to identify patients with SMA and for severity stratification, potentially complementing current diagnostic and prognostic strategies in SMA management.

## 1. Introduction

5q spinal muscular atrophy (SMA) is an autosomal-recessive motor neuron disease caused by the bi-allelic loss of function of the survival of motor neuron 1 (*SMN1*) gene on chromosome 5q13 with at least one functional copy of the paralogous survival of motor neuron 2 (*SMN2*) gene. The clinical SMA spectrum is diverse, ranging from severely affected patients showing pre- or early postnatal onset to more attenuated phenotypes with variable disease [1]. Traditionally, childhood-onset SMA has been divided into SMA 1 (“non-sitters”) with disease onset between 0 and 6 months, SMA 2 (“sitters but non-walkers”) presenting between 7 and 18 months, and SMA 3 (“walkers”) becoming clinically apparent before (3a) or after (3b) 3 years of age [2,3]. Recently, the development of novel disease-modifying therapies has dramatically changed the disease trajectories of SMA. Currently, three highly effective therapeutics are available, including the antisense oligonucleotide Nusinersen, the small molecule compound Risdiplam, and the gene addition therapy (GAT) Onasemnogene abeparvovec, all increasing functional SMN protein levels, preserving motor function, and improving life expectancy and quality of life [4,5].

To shorten the administration time of disease-modifying treatments [6,7,8,9], recent implementations of newborn screening programs for SMA have enabled clinicians to identify affected individuals during an early or even presymptomatic disease stage and to initiate timely treatment [10,11,12].

Despite these advances, stratifying individuals with a positive newborn screening result remains a major challenge due to the lack of definitive biomarkers needed to predict disease severity. This problem in patient stratification includes early-onset cases, which require immediate treatment or bridging strategies until definitive interventions are available, and later-onset cases, which may benefit from clinical monitoring [13]. Consequently, a number of promising approaches have been taken to predict individual courses of disease, including *SMN2* copy numbers, SMN-transcript and protein levels, plasma phosphorylated neurofilament heavy chain, compound muscle action potential (CMAP) measurements, and multi-omics approaches [14,15,16,17,18,19,20,21,22,23]. *SMN2* copy numbers and CMAP measurements are widely used in routine care; however, they have failed to show a sufficiently high prognostic value [13,24]. *SMN2* copy numbers, despite currently being the main determinant for therapeutic decisions in SMA, hold limited prognostic value regarding disease progression and treatment response, are often difficult to quantify, and have shown inconsistencies between predicted and observed phenotypic outcomes, especially for individuals harboring three copies [25,26]. Conversely, standardized electrophysiological examinations have demonstrated utility as indicators of symptom onset in SMA. However, they present notable challenges in newborns, including high interrater variability, technical challenges in obtaining reproducible results, and a lack of consensus guidelines regarding the optimal timing of investigations and locations for stimulation [27,28].

The clinical phenotype of SMA is influenced by various intrinsic (genetic and metagenetic) and extrinsic (environmental) factors. Adopting a comprehensive approach that incorporates a wider array of biological information might substantially enhance clinical decision making. Among the various omics technologies, metabolomics harbor considerable potential to capture the intricate complexities inherent in a living system. By integrating intrinsic and extrinsic factors, metabolomics provides an accurate, dynamic, and comprehensive picture of an individual’s state of health and progression of disease and has demonstrated high correlations with clinical phenotypes in several neurologic diseases [29,30]. Thus, metabolic profiling bears the potential to shed light on the pathophysiological processes occurring in SMA and might support clinicians in the decision on how to proceed after a positive newborn screening result. One of the most widely used techniques for metabolic profiling is nuclear magnetic resonance (NMR) spectroscopy. While NMR has lower sensitivity and resolution compared to mass spectrometry, another key method in metabolomics, it offers several advantages. NMR is non-selective and non-destructive, requires minimal sample preparation, allows for the analysis of intact biofluids, detects a wide range of metabolites simultaneously, and produces highly reproducible and quantifiable results, making it an efficient high-throughput analytical tool [31].

We previously investigated the potential of ^1^H-NMR urinary metabolic profiling for SMA diagnosis and severity prediction in a pilot study involving 24 patients [21]. In this study, we further investigate the biochemical changes underlying SMA pathology and determine SMA subtypes using ^1^H-NMR spectroscopy-based metabolic profiling in different biofluids in a multicentric real-life cohort. Our results might serve as a potential starting point to develop a modality for the confirmation of an SMA diagnosis and the prediction of disease severity.

## 2. Results

### 2.1. Study Cohort

A total of 213 biosamples (N_urine_ = 116, N_plasma_= 38, and N_CSF_ = 59) from 153 symptomatic treatment-naïve patients with SMA (N_G12.0_ = 56, vs. N_G12.1_ = 97) from the MetabNMD study were analyzed. The patient and biomaterial contributions from each center are shown in Table S1. Complete baseline characteristics were available for 76% of patients (Table 1). The median age at sample collection was 10.08 years (IQR 22.33, range: 0–67). The median age in the severely affected G12.0 group was significantly lower than that in the mildly to moderately affected G12.1 group (median_G12.0_ 1.04 (IQR 7.72) years vs. median_G12.1_ 17.63 (IQR 22.04) years; Mann–Whitney U test, W = 851, *p* < 0.001; see in Supplementary Figure S1). The age bias was reduced using age-matched controls whenever possible. The male-to-female ratio was roughly 1:1 in all groups. As expected, and in line with previous publications [12], most individuals (75%) in the G12.0 group harbored two *SMN2* copies, while in the G12.1 group, the majority of individuals had three (59.8%) or four (32%) *SMN2* copies. The relation of patients with SMA II to patients with SMA III in the G12.1 group was roughly 1:1.5. The CHOP INTEND scores in both groups were comparable, with the limitation that only the most severely affected individuals in the G12.1 group, e.g., due to advanced disease, underwent this test. The HFMSE scores were higher in the patients in the G12.1 cohort compared to the G12.0 cohort (mean_G12.0_ 8.5 ± 2.12 vs. mean_G12.1_ 25.83 ± 20.26); however, a statistical analysis was not performed due to small sample sizes.

The results of the collection and analysis of samples from healthy controls for the urine cohort have been published previously in parts [23]. Additional healthy urinary samples were added in this study to increase the age range for the healthy controls. The controls for CSF were established during diagnostic sample collections (e.g., children undergoing a lumbar puncture for the evaluation of facial palsy, suspected meningitis, etc., without pathological findings in the work-up). The plasma controls were collected from healthy individuals as part of this study. The healthy control cohort resembled the SMA cohort regarding age (median_SMA_ 10.08 years (IQR 22.33, range 0–67) vs. median_Healthy_ 5.00 years (IQR 11.36, range 0–67). The age distribution among the different body fluids is shown in Supplementary Figure S1.

### 2.2. ^1^H-NMR Metabolic Profiles of Urine, Plasma, and CSF Show Clear Separation Between Patients with SMA and Healthy Controls

**Figure 1:**
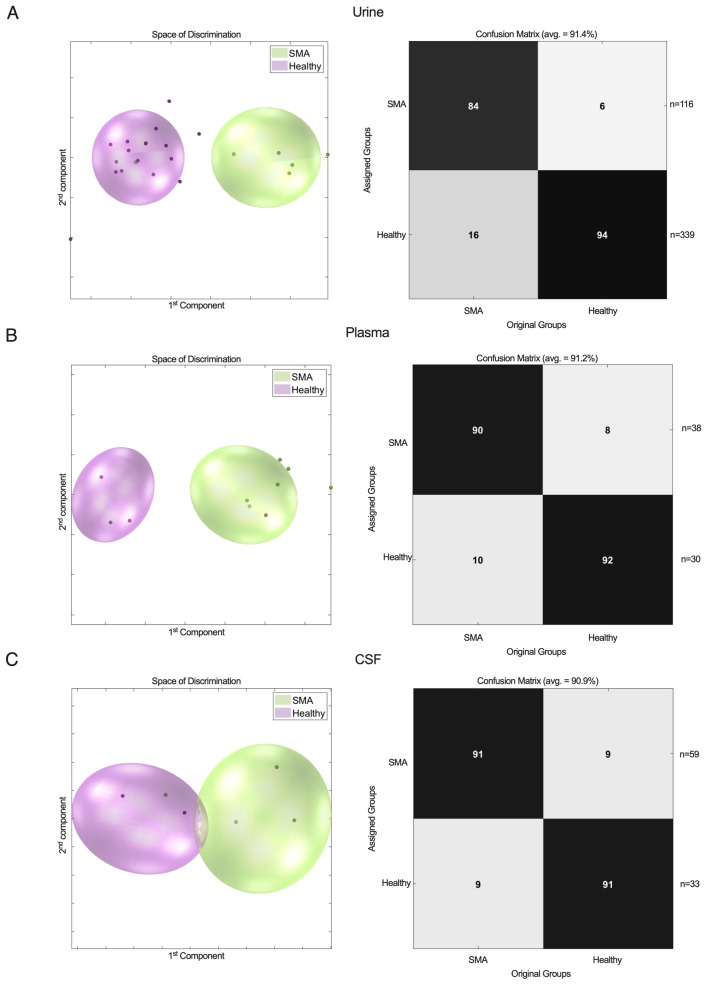
^1^H-NMR metabolic profiles in urine (**A**), plasma (**B**), and CSF (**C**) from patients with SMA vs. healthy controls. PCA/CA classification and MCCV showed clear discrimination between SMA group and healthy control group.

The urinary samples from the severely (G12.0, SMA type I, *n* = 39) and mildly to moderately (G12.1, SMA type II/III, *n* = 77) affected patients with SMA were pooled and tested against a healthy control group (*n* = 339). The PCA/CA/k-NN classification of the metabolic profiles in urine (N_SMA_ = 116, vs. N_HEALTHY_ = 339) showed good separation with a sensitivity of 84% and a specificity of 94% (Figure 1A).

To extend these findings to other biofluids and determine the ideal medium for metabolic profiling, we next analyzed plasma (N_SMA_ = 38 [N_G12.0_ = 8; N_G12.1_ = 30] vs. N_HEALTHY_ = 30) and CSF (N_SMA_ = 59 [N_G12.0_ = 25 vs. N_G12.1_ = 34] vs. N_HEALTHY_ = 33) samples. After confirming the findings in urine, the patients with SMA were identified with a comparable accuracy of >90% (Figure 1B,C).

Together, these findings suggest that all three biomaterials show adequate properties and comparable accuracy for identifying patients with SMA vs. healthy controls using ^1^H-NMR-based metabolic profiling.

### 2.3. ^1^H-NMR Metabolic Profiles of Urine, Plasma, and CSF Can Predict SMA Disease Severity

After showing that individuals with SMA can be robustly distinguished from healthy individuals, we next analyzed whether the ^1^H-NMR profiles were able to reflect disease severity. Patients with SMA were divided into early and severely affected (G12.0, SMA type I) vs. mildly to moderately affected groups (G12.1, SMA type II/III) according to the latest ICD-10 classification. Since, from a clinical standpoint, there is a particular need for biomarkers identifying early-onset (SMA type I) patients who need immediate treatment from later-onset (SMA type II and III) patients, and since clinical distinction between type II and III patients is sometimes challenging, reflecting a disease continuum rather than separate entities, we refrained from further subdividing the G12.1 group. Biomaterial was collected to establish a urine cohort (N_G12.0_ = 39 vs. N_G12.1_ = 77 vs. N_HEALTHY_ = 339) as well as cohorts for plasma (N_G12.0_ = 8 vs. N_G12.1_ = 30 vs. N_HEALTHY_ = 30) and CSF (N_G12.0_ = 25 vs. N_G12.1_ = 34 vs. N_HEALTHY_ = 33).

**Figure 2:**
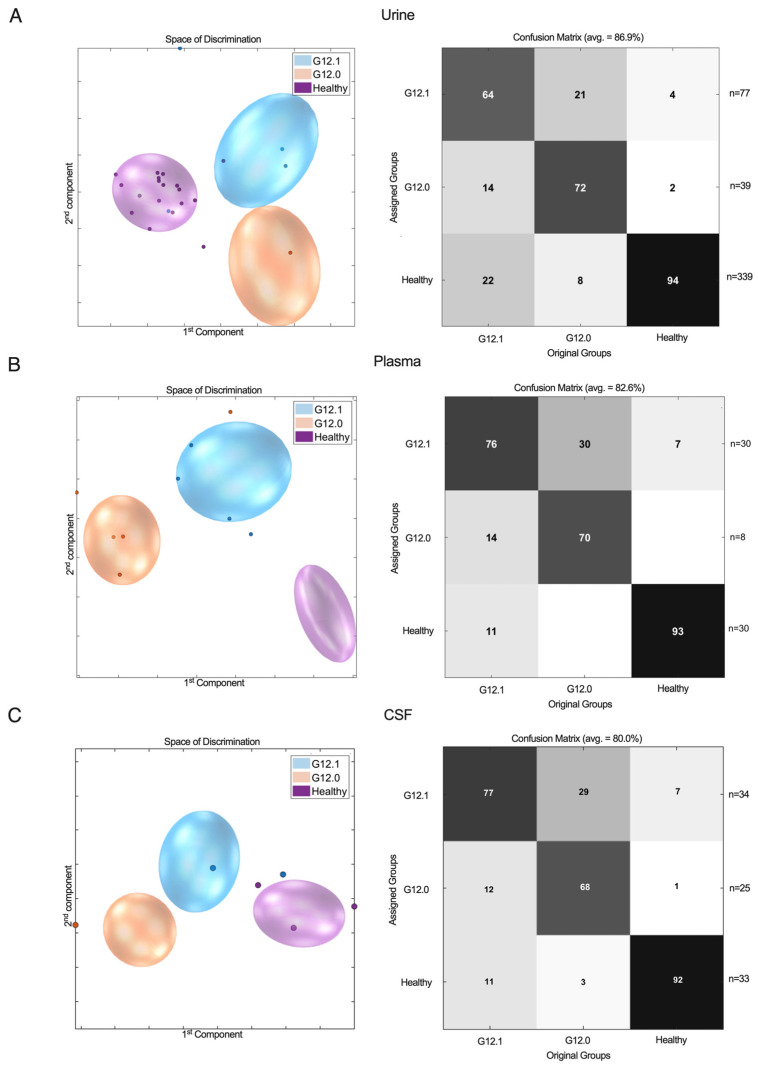
^1^H-NMR metabolic profiles of G12.0 (severely affected) and G12.1 (mildly to moderately affected) vs. healthy controls in urine (**A**), plasma (**B**), and CSF (**C**) samples. PCA/CA classification and MCCV showed clear discrimination between severity subgroups and the healthy control group.

The healthy controls were correctly classified in all biomaterials with high sensitivity and a specificity in the range of 86–94% (Figure 2). False assignment to the mildly to moderately affected cohort occurred in a few cases (4% of urine samples, 7% of plasma samples, and 7% of CSF samples). Erroneous classification of healthy individuals and severely affected G12.0 patients was rarely observed (2% of urine samples, 1% of CSF samples, and none of the plasma samples). The severely affected G12.0 group was correctly classified in roughly two-thirds of cases across all biomaterials (urine: 72%, plasma: 70%, and CSF: 68%) and showed overlap with the G12.1 group in 21% (urine), 30% (plasma), and 29% (CSF) of cases. False assignment of G12.0 cases to healthy controls rarely occurred in the urine samples (8%) and in CSF samples (3%) and was not observed for plasma.

Similarly, for the G12.1 cohort, 64% (urine), 76% (plasma), and 77% (CSF) of patients were correctly assigned to their groups, while overlap with the healthy cohort occurred in 22% (urine), 11% (plasma), and 11% (CSF) of cases. False classification to the G12.0 group was equally rare (14% for urine and plasma and 12% for CSF).

### 2.4. Protein Signal Plays a Major Role in SMA Subgroup Differentiation

The untargeted PCA/CA/k-nn/MCCV classification approach described above, also known as the top-down strategy, bypasses the need for a predetermined a priori hypothesis regarding the selection of a specific set of metabolites, but instead examines the entire metabolic profile. Therefore, untargeted metabolomics can also be regarded as a “discovery mode” based on differential comparisons between study groups.

**Figure 3:**
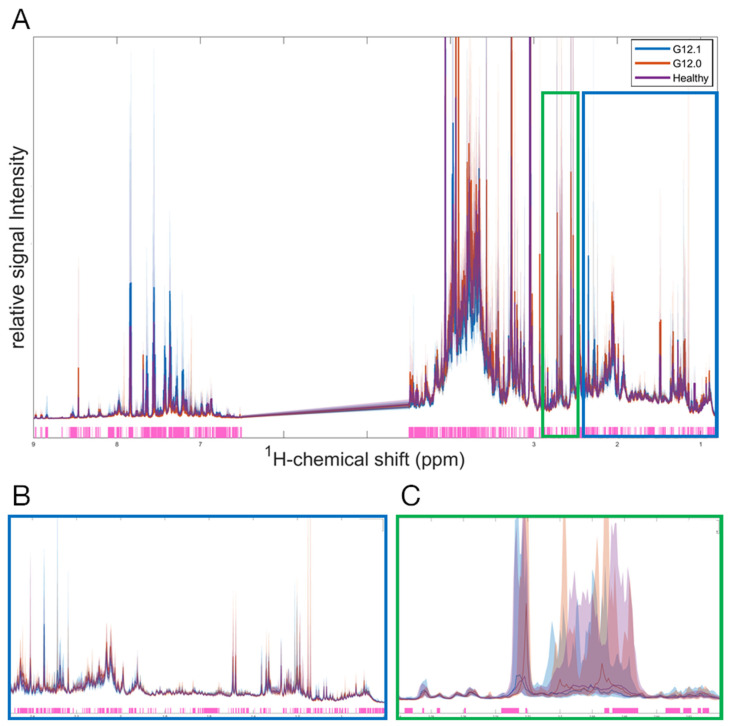
A univariate analysis of the full ^1^H-NMR urinary spectrum for G12.0, G12.1, and the healthy control group. The discriminating region between the groups revealed by the Kruskal–Wallis test (at *p* < 0.05 significance level) is highlighted in light pink. The median of each group is represented by a line (G12.0 is red, G12.1 is blue, and the healthy control is purple), and the 5–95% percentile of each group is represented by the corresponding light color area. (**A**) full ^1^H-NMR urinary spectrum (0.8–9.0 ppm). (**B**) A zoomed in figure of the aliphatic region (0.8–2.5 ppm). (**C**) A zoomed in figure of a specific region illustrating the discrimination between all 3 groups (2.6–2.8 ppm).

Statistical differences between the study groups were evaluated by applying the Kruskal–Wallis H-test, revealing significant differences (*p* < 0.05) in the SMA subgroups and healthy controls across the ^1^H-NMR spectra in all three biofluids. In urine, significant differences were found in both the aliphatic and aromatic regions (Figure 3). Overall, 69% of the analyzed urinary spectral regions showed significant differences between at least two groups, with 46% of these differences involving metabolites or proteins that have not been annotated and attributed to quantified substrates yet. Similarly, significant differences among subgroups were found in 76% of plasma and 72% of CSF profiles, with 32% and 52% not yet being attributed to quantified metabolites, respectively.

### 2.5. Quantitative Enrichment Analysis Reveals Distinctive Differences in Energy and Amino Acid Metabolism Among SMA Subtypes

**Figure 4:**
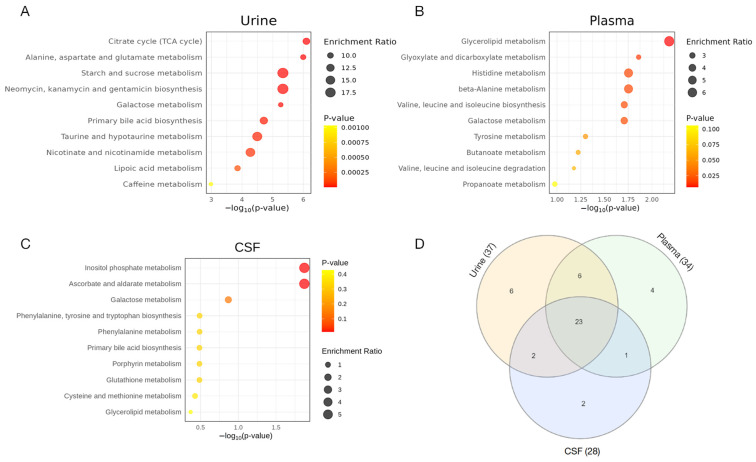
A summary plot for the quantitative enrichment analysis of G12.0 vs. G12.1 in patients’ urine (**A**), plasma (**B**), and CSF (**C**) samples. The quantitative enrichment analysis was performed by mapping metabolites on the KEGG human database using Metaboanalyst 6.0. The top 25 enriched metabolite sets are plotted. The X-axis represents the −log(*p*-value) indicating the significance of pathway enrichment, while the Y-axis lists the affected metabolic pathways. The dot size reflects the enrichment ratio. (**D**) Additionally, a Venn diagram is included to visualize the overlap between enriched pathways. The metabolite sets that overlap between all three sample qualities are the citrate cycle (TCA cycle); alanine, aspartate, and glutamate metabolism; glyoxylate and dicarboxylate metabolism; starch and sucrose metabolism; neomycin, kanamycin, and gentamicin biosynthesis (D-Glucose); primary bile acid biosynthesis; galactose metabolism; cysteine and methionine metabolism; valine, leucine, and isoleucine biosynthesis; lipoic acid metabolism; glutathione metabolism; porphyrin metabolism; glycine, serine, and threonine metabolism; pyruvate metabolism; glycolysis/gluconeogenesis; pantothenate and CoA biosynthesis; phenylalanine, tyrosine, and tryptophan biosynthesis; phenylalanine metabolism; arginine and proline metabolism; butanoate metabolism; tyrosine metabolism; valine, leucine, and isoleucine degradation; and glycerophospholipid metabolism.

**Figure A1:**
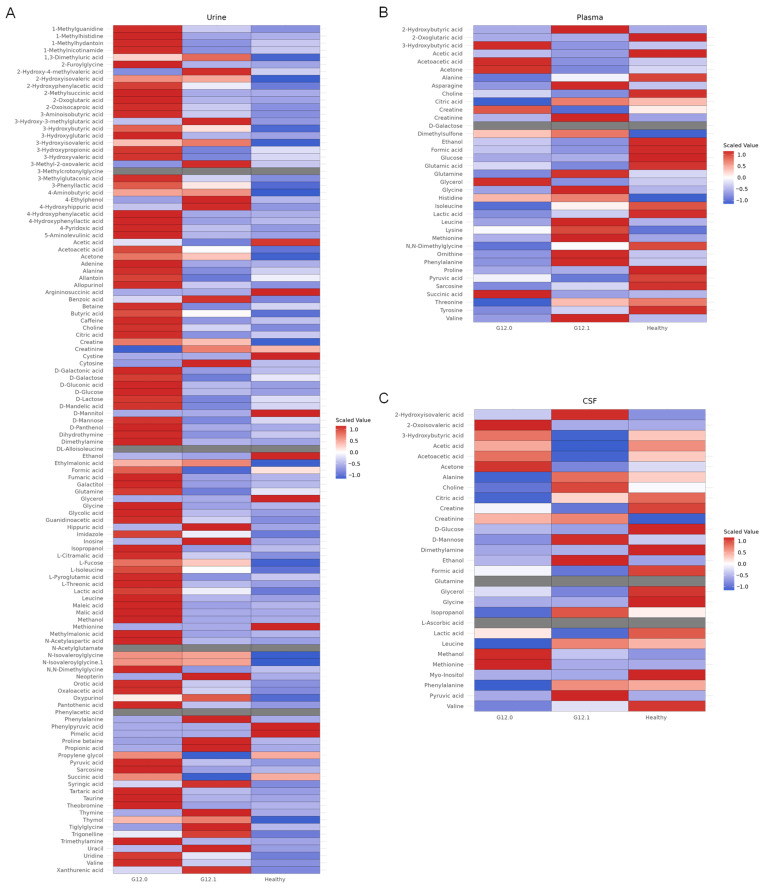
Visualization of metabolite levels in G12.0, G12.1, and healthy controls in different biomaterials, represented by heatmaps showing group average. Red indicates high levels compared to mean value; blue indicates low levels compared to mean value. Gray indicates missing values. (**A**) Urine, (**B**) plasma, and (**C**) CSF.

Despite lacking annotations of large parts of the ^1^H-NMR spectra, the mean metabolite concentrations in the well-annotated regions showed minor but relevant differences among groups in all biofluids (Figure A1). To explore differentially enriched metabolic pathways in the patients with SMA vs. healthy controls, as well as the SMA subtypes, we performed a quantitative enrichment analysis (QEA) among the annotated metabolites in all three biofluids [32]. The top 10 enriched metabolite sets distinguishing severely from the mildly to moderately affected patients with SMA are shown in Figure 4A–C. Interestingly, changes in the urinary spectra showed the most pronounced differences in separating early- from late-onset SMA. Overlaps of enriched metabolite sets across all biofluids were merged in a Venn diagram (Figure 4D). A total of 23 metabolic pathways were identified as being altered between the SMA severity groups in the QEA among all three biomaterials, mainly involving energy and amino acid metabolism. The complete QEA results for G12.0 vs. G12.1 are shown in Supplementary Material Table S2.

## 3. Discussion

Since the development of disease-modifying therapeutics and the successful implementation of newborn screening programs for SMA in a growing number of countries, the life expectancy and disease trajectories of patients with SMA have dramatically improved. However, the timely initiation of therapy remains a critical challenge given the cost, invasiveness, and safety concerns associated with these novel treatments. Despite notable progress in genetics and biomarker research, differentiating between early- and late-onset patients and developing reliable diagnostic algorithms remain clinically important challenges. In particular, the identification of children who are at risk for early symptom onset and a severe disease course requiring imminent treatment initiation remains an unmet need.

In a previous pilot study, we demonstrated that ^1^H-NMR spectroscopy-based metabolic profiling is a feasible and effective method for the diagnosis and prediction of clinical phenotypes in urine samples from 24 treatment-naïve symptomatic individuals with SMA [23]. In this study, we extended our cohort to analyze urine, plasma, and CSF samples from 153 treatment-naïve symptomatic patients with SMA. Across all biomaterials, we were able to generate specific metabolic profiles from treatment-naïve symptomatic patients.

Our prediction models show robust discrimination between patients with SMA and healthy controls in approximately 90% of cases. Even though the sample sizes differed considerably between the urine, plasma, and CSF cohorts, the accuracy of our predictions was comparable across all biomaterials, suggesting that disease-specific changes in SMA may affect multiple organ systems in line with previous studies demonstrating ubiquitous SMN expression in all tissues [33]. Since SMA is increasingly recognized as a multisystem disease and the loss of SMN appears to lead to the disruption of intricate molecular networks [34], the systemic effects resulting in SMA pathology might be more accurately reflected in urine and plasma than in CSF.

From a clinical perspective, no biomaterial appears to be superior to the other. We suggest that urine, which is a non-invasive and easily accessible medium, is an optimal choice for clinical translation. However, plasma, which is obtained during clinical diagnostics and is non-inferior, may also be a suitable biomaterial for the clinical validation of our models.

To address the clinical challenge of differentiating between severely and mildly to moderately affected individuals, disease severity models were created in the second step. Patients were assigned to a severely affected group (G12.0, SMA type I) or a mildly to moderately affected group (G12.1, SMA type II/III). This simplification was made to reduce differences in age between groups and reflects the clinically relevant risk stratification. Influences due to medication were eliminated by including only treatment-naïve samples in our analysis.

The metabolic profiles in all biomaterials demonstrated reliable recognition of healthy individuals and an overall good recognition of severe SMA cases. As expected, given the fact that SMA is clinically and biochemically a disease continuum with borderline phenotypes, there was a 10–20% overlap of the mildly to moderately (G12.1) affected group with both severe cases (G12.0) and healthy controls. Interestingly, those cases from the G12.1 group that were misassigned to the healthy control group showed a tendency to depict higher scores in motor function tests, although these differences did not reach significance. Conversely, a few healthy individuals were assigned to the mildly to moderately affected group. We hypothesize that this could be due to a heterozygous *SMN1* carrier status; however, we are currently unable to prove this hypothesis.

To explore the potential metabolic pathways responsible for the differences between patients with SMA and healthy controls, as well as between different SMA subtypes, we conducted a quantitative enrichment analysis on our identified metabolite sets. Significant differences in arginine and proline metabolism were observed between the patients with SMA and healthy controls, which can be attributed to the unspecific but highly altered creatine/creatinine ratios. Among all biomaterials, the metabolite sets involved in energy and amino acid metabolism showed significant differences, which is consistent with previous data from patients with SMA and animal models [35,36]. Additionally, a recent study by Errico et al. showed alterations in similar pathways induced by Nusinersen treatment in CSF samples [37], underscoring their potential role in SMA pathophysiology. Along these lines, resting energy expenditure has been found to be elevated in patients with SMA type I, highlighting the altered energy metabolism in SMA types and suggesting a potential overall increase in catabolic pathways in severe cases. The differences in energy metabolism between early- and late-onset patients with SMA were the most pronounced in the urinary metabolome. Interestingly, urine has previously been proposed as the preferred medium for biomarker development in neurologic diseases due to its lack of homeostatic mechanisms that might attenuate subtle systemic fluctuations reflective of disease onset and progression [38,39].

Overall, the differences in the metabolite sets observed in our QEA were within the range of one standard deviation, with significant overlap between the groups, therefore providing limited use as potential biomarkers. However, a significant amount of information contained in the spectra can currently not be included in this approach due to a lack of annotation. This particularly involves large regions of protein background, including the aliphatic and aromatic regions of the spectrum where the major differences between subgroups of patients with SMA and healthy individuals seem to reside. Further in-depth investigations of these non-annotated areas of the spectrum, including the use of complementary omics approaches, might provide future insights into the pathophysiology of SMA and allow for developments of more specific biomarkers. Until then, an untargeted approach appears to be more beneficial, taking into account the entire information present in a biological sample. Systems biology approaches in mouse models of SMA have already begun to unravel the complex molecular networks interacting with the SMN protein and identified potential molecular regulators that might serve as therapeutic targets [36].

Together, our findings underline the potential benefits of using untargeted ^1^H-NMR spectroscopy-based metabolic profiling as an additional tool complementing current stratification strategies, such as *SMN2* copy numbers and CMAP-testing, in predicting the severity of patients with SMA. Given its minimal preanalytical requirements and time-efficient measurement, the implementation of ^1^H-NMR spectroscopy may be feasible and beneficial in a time-critical clinical setting. The results provided in this study might help in increasing understanding of the complex pathophysiology of SMA and offer guidance in future clinical trials and therapeutic decision making.

We acknowledge several limitations to our approach, with some being specific to our study design and others being inherent to NMR spectroscopy. First, our results may be biased by varying sample sizes across sub-cohorts and the limited availability of treatment-naïve biosamples for different age groups. The implementation of several disease-modifying therapies along with newborn screening programs during the recruitment period of this study naturally limited the availability of treatment-naïve symptomatic patients with SMA to pre-screening cohorts. However, despite these challenges, our predictions remained consistently robust across all biomaterials, underscoring the overall reliability of our approach. Second, while we were able to identify highly significant changes in core metabolic pathways within well-annotated regions of the ^1^H-NMR spectra, a substantial portion of the spectra contributing to the differences between patients with SMA and healthy controls, as well as SMA subtypes, comprise a non-annotated protein background, which is currently beyond the scope of our method. We show that despite this inherent limitation of NMR spectroscopy, an untargeted approach can accurately predict SMA disease severity. Nevertheless, elucidating the precise proteomic and metabolomic changes underlying SMA phenotypes across multiple cell lines and disease models, including patient-derived iPSC-neurons or brain organoids, would be desirable to fully understand the molecular mechanisms of disease progression. Along these lines, a bedside-to-bench approach using complementary techniques in SMN-deficient mouse models has recently confirmed and further characterized some of the biochemical alterations in SMA reported in this study [34].

In conclusion, we showed that metabolic profiling using ^1^H-NMR spectroscopy is a suitable “fingerprinting” method for untargeted diagnosis and disease severity prediction in a real-life multicenter SMA cohort. Despite the multifaceted influences on the metabolome, we were able to develop models suitable for the diagnosis and severity prediction of SMA. Our work highlights the potential for translating ^1^H-NMR metabolic profiling into clinical practice. Even outside of highly controlled conditions and with a diverse clinical cohort, the chosen approach yielded promising results. Together with other genetic (e.g., *SMN2* copy number, modifier genes), clinical (e.g., CHOP INTEND at baseline), and electrophysiological (e.g., CMAP) parameters, our method might help to establish a reliable toolbox for predicting early disease onset and therefore the necessity for immediate treatment initiation. Especially in milder phenotypes and children with four *SMN2*-copies diagnosed via newborn screening, this marker set might help to determine optimal time windows for treatment initiation.

Future research projects might address the questions of whether ^1^H-NMR spectra can predict disease onset and severity in pre-symptomatic children identified through newborn screening and to analyze whether dynamic changes in metabolic profiles under therapy might predict treatment responses. Collectively, ^1^H-NMR-based metabolic profiling identified relevant metabolic differences between healthy individuals and patients with SMA and revealed potential biochemical determinants of SMA disease progression. Our findings point to the presence of complex interaction networks involved in SMA pathology that might serve as a starting point to develop risk–benefit assessment tools or even personalized treatment strategies for individuals affected with SMA and related neurogenetic disorders.

## 4. Materials and Methods

### 4.1. The MetabNMD Study

This work comprises data and samples from the MetabNMD study (study reference: EUPAS32033). In this multicenter longitudinal observational study, biomaterials (urine, plasma, and CSF) from 202 individuals with genetically confirmed SMA were collected from five participating centers (University Hospitals Essen, Giessen, Hamburg, Heidelberg, and Munich) in Germany between 2018 and 2023.

For this study, biomaterials from 153 treatment-naïve patients were selected. A total of 116 urine, 59 CSF, and 38 plasma samples were analyzed. A total of 402 healthy individuals were used as the control cohort as reported previously [23].

### 4.2. Clinical Data

Clinical data were collected during routine inpatient and outpatient visits. Data analyzed included age, sex, *SMN2* copy number, SMA subtype, and CHOP INTEND and HFMSE motor scores. SMA subtypes were grouped into two main categories according to the International Statistical Classification of Diseases and Related Health Problems (ICD-10) [40]:

(1) G12.0: SMA type I (“severe”);
(2) G12.1: SMA type II/III (“mild to moderate”).

### 4.3. Sample Collection and Preparation

Standard operating procedures (SOP), based on the technical specification DIN CEN/TS 16945:2016-11 [41], were created for sample collection, preanalytical processing, storage, and shipment. Samples were processed in the respective centers and stored in the NCT Cell and Liquid Biobank, Heidelberg, Germany, at −80 °C. All shipments were sent on dry ice, and temperature was constantly monitored.

Whenever possible, midstream urine was obtained, either spontaneously or as a “clean catch” sample. Whenever midstream sampling was impossible (e.g., young children or individuals with impaired bladder control), bagged urine was obtained. A urinary point-of-care test for leucocytes, blood, glucose, and proteins was performed, the results were documented, and the urine was transferred to a sample tube. Samples were stored at room temperature for a maximum of 30 min and for 6 h at 4 °C prior to processing. Urine samples were centrifuged at 4 °C and 2500× *g* for 5 min. Subsequently, supernatant was pipetted into 2 mL cryovials and stored at −80 °C. Urine with a pathological point-of-care diagnostic was excluded from the analysis. Cerebrospinal fluid (CSF) was obtained during a routine diagnostic procedure or prior to intrathecal medication application. An amount of 2 mL of CSF was collected according to clinical standards. CSF was stored at room temperature for a maximum of 30 min. Subsequently, CSF was centrifuged at 4 °C and at 2500× *g* for 5 min, and the supernatant was aliquoted to 750 μL cryovials and stored at −80 °C. CSF with procedural blood contamination was excluded from the analysis. An amount of 2.7 or 9 mL of venous blood was taken during routine diagnostic procedures using K^+^-EDTA tubes according to clinical standards. EDTA blood was stored for a maximum of 30 min at room temperature or 4 h at 4 °C prior to processing. EDTA blood was centrifuged at 2500× *g* at room temperature for 10 min. Plasma was separated from buffy coat and red blood cells and stored at −80 °C.

Urine, plasma, and CSF samples were prepared according to standard procedures as described previously [42,43]. Frozen urine, plasma, and CSF samples were thawed at 4 °C and shaken before use. An amount of 0.9 mL of urine was added to 0.1 mL of potassium phosphate buffer (pH 7.4) containing trimethylsilyl propionic acid-d4 sodium salt (TSP) and sodium azide, and 0.35 mL of plasma was added to 0.35 mL of sodium phosphate buffer (pH 7.4) containing trimethylsilyl propionic acid-d4 sodium salt (TSP) and sodium azide. An amount of 0.75 mL of CSF was added to 0.6 mL of buffer. The mixture, either urine, plasma, or CSF, was homogenized, and 0.6 mL was transferred to a 5 mm NMR tube and placed in a cooled sample changer for analysis.

### 4.4. ^1^H-NMR Spectroscopy

All samples were measured in full automation according to standard procedures on a Bruker IVDr System, as described previously [42]; a 600 MHz Bruker Avance III HD or Avance NEO NMR spectrometer equipped with a SampleJet sample changer with sample cooling and a pre-heating station, a 5 mm inverse probe with z-gradient and automated tuning and matching, and a BCU-I TopSpin 3.6 or higher cooling unit in combination with Bruker’s body fluid NMR method package B.I.Methods 2.0/2.5 were used for fully automated acquisition and processing controlled by ICON NMR (Bruker Biospin GmbH, Ettlingen, Germany).

Prior to measurement, samples were kept for 5 min inside the NMR probe head for temperature equilibration at 27 °C (300 K) for urine and CSF and at 37 °C (310 K) for plasma. Tuning and matching, locking, shimming, the optimization of the lock phase, and the calibration of the hard pulse at 90 °C were carried out automatically for the optimization of the NMR experimental conditions. Next, one-dimensional ^1^H-NMR NOESY (Nuclear Overhauser Effect SpectroscopY, 32-scan) spectra were acquired by applying a standard pulse sequence with suppression of the water signal (Bruker pulse program library noesygppr1d, Bruker Biospin GmbH, Ettlingen, Germany). Fourier transformation and fully automated phasing and baseline correction were carried out via the Bruker standard automation program APK0.NOE (Bruker Biospin GmbH, Ettlingen, Germany). Spectra were quantitatively calibrated via the PULCON principle using an external reference sample [44]. TSP (Trimethylsilyl propionic acid-d_4_ sodium salt) served as the reference peak. Two-dimensional ^1^H-NMR JRES (J-RESolved spectroscopy, 2-scan) spectra were acquired. For plasma, CPMG (Carr–Purcell–Meiboom–Gill, 32-scan), DIFF (DIFFusion, 32-scan), and PGPE (Pulsed Gradient Perfect Echo, 64-scan) spectra were run.

### 4.5. Spectral Analysis and Binning

**Urine:** Spectral intensity was scaled using a minimum baseline scale using the 1.0–4.3 ppm region to eliminate any variability coming from spot urine. Afterward, each spectrum was segmented from 0.8 to 9 ppm into consecutive bins containing 0.01 ppm, and the pertaining regional integrals (bin intensities) excluding the 4.5–6.5 ppm region were calculated.

**CSF:** Spectral intensity was scaled to a mmol/L concentration scale. Afterward, each spectrum was segmented from 0.8 to 4.55 ppm into consecutive bins containing 0.02 ppm, and the pertaining regional integrals (bin intensities) excluding the 4.5–6.5 ppm region were calculated.

**Plasma:** Spectral intensity was scaled to a mmol/L concentration scale. Afterward, each spectrum was segmented from 0.3 to 10 ppm into consecutive bins containing 0.01 ppm, and the pertaining regional integrals (bin intensities) excluding the following regions were calculated: 3.04–3.31 ppm, 3.59–3.66 ppm, and 4.5–5.5 ppm. The exclusion of the named bins was necessary to remove biases from irrelevant sample variability, i.e., water signals present in all biofluids and EDTA signals present in plasma samples. A so-called bucket table was created, with rows representing samples and columns representing bins.

### 4.6. Multivariate Analysis

**Principal component analysis (PCA):** PCA was used as an unsupervised multivariate technique for coordinate transformation on the initial bucket table to separate relevant signals from noise. It projects correlated variance distributed over several variables onto single new variables, the Principal Components, thus simplifying visualization and interpretation. In this study, dimensionality reduction and data visualization were carried out by PCA, and data tables were prepared for further multivariate analyses.

**PCA/CA/k-NN Classification:** After dimensionality reduction in the bucket table by a PCA, a canonical analysis (CA) in combination with a MANOVA was performed to determine the subspace for maximum class separation and its respective dimension. A classification system was introduced using the k-nearest neighbor (k-NN) concept. Together, the PCA/CA/k-NN classification procedure is a supervised method to first project a new sample into the PCA/CA subspace and then assign its class by k-NN.

**Monte-Carlo embedded cross-validation (MCCV):** As PCA/CA/k-NN classification is a supervised method, related models are established with known class membership for each sample. Therefore, an extensive validation is necessary to avoid overfitting the model during the training phase. In this study, MCCV was used to optimize the correct classification. A confusion matrix with n × n fields (with n representing the number of classes to be discriminated) was created from MCCV, where diagonal fields represent the probability of true classification and off-diagonal fields relate to probabilities of misclassification, e.g., samples of class A being falsely assigned to a different class, such as B.

PCA/CA/k-NN classification and MCCV are further described by Assfalg et al. and Bernini et al. [45,46]. The prediction of sample classes in the discriminatory space was carried out by selecting the lowest distance to the center of the group.

### 4.7. Univariate Analysis

We employed the Kruskal–Wallis H test to identify significant differences among the SMA groups. Each bin’s intensity was treated as an independent observation. The Kruskal–Wallis test compared the median intensities of these bins across the different groups to assess spectral bins showing statistically significant variations between groups.

### 4.8. Quantitative Enrichment Analysis

Quantification results of 150 urinary metabolites were obtained directly after the NMR measurement using the Bruker IVDr Quantification in urine for children and adults (B.I.QuantUR-e 1.1, Bruker Biospin GmbH, Ettlingen, Germany) module. The concentrations of the 112 lipoproteins and subclasses, the 40 small molecules, and the 5 inflammatory analytes were obtained by analyzing the plasma spectra using the Bruker IVDr Lipoproteins and Subclasses analysis (B.I.LISA 1.0, Bruker Biospin GmbH, Ettlingen, Germany) module, the Bruker IVDr Quantification in plasma (B.I.QuantPS2.0, Bruker Biospin GmbH, Ettlingen, Germany) module, and the PhenoRiskPACS module(Bruker Biospin GmbH, Ettlingen, Germany), respectively. Lipoproteins were not included in the enrichment analysis. The concentration of the 29 analytes in CSF was obtained using a Bruker in-house quantification algorithm. To provide a comprehensive visualization of metabolite concentrations, heatmaps using normalized data and the Euclidian distance between metabolites were generated (Figure 4) [47]. A metabolite set enrichment analysis was conducted using MetaboAnalyst 6.0 [32]. Urinary metabolite concentrations were normalized to a relative concentration presented in mmol/mol creatinine and auto-scaled by mean-centering and division by the standard deviation of each metabolite. Plasma and CSF metabolites (absolute concentration mmol/L) were mean-centered and divided by the standard deviation of each metabolite. Metabolic pathways were annotated using the KEGG Metabolic pathways *Homo sapiens* database [48]. Globaltest was used to calculate the Q-statistics for each metabolite set, describing the correlation between the concentration profiles and clinical outcomes in a linear model [49]. A Venn diagram was used to visualize overlaps in the enriched metabolite sets.

### 4.9. Baseline Statistics

Baseline characteristics were summarized using either the mean and standard deviation (SD) or median and interquartile range (IQR) or median and min-max description depending on the distribution of data tested by visualization with histograms, quantile-quantile plots, and normality testing using the Shapiro–Wilk test. Sample sizes are indicated (n) for each analysis. The *t*-test (for normally distributed variables) and the Mann–Whitney U and Kruskal–Wallis tests (for non-parametric distributions) were performed to test for statistical differences.

## Figures and Tables

**Table 1:** Demographic characteristics of study participants.

		Overall SMA (*n* = 153)	G12.0 (*n* = 56)	G12.1 (*n* = 97)	Healthy Controls (*n* = 402)
Age [years] (median, IQR)		10.08 (22.33)	1.04 (7.72)	17.63 (22.04)	5.00 (11.36)
Sex (*n*, %)	unreported	2 (1.3%)	-	2 (2.1%)	-
	female	81 (52.9%)	30 (53.6%)	51 (52.6%)	181 (45.0%)
	male	70 (45.8%)	26 (46.4%)	44 (45.4%)	221 (55.0%)
SMN2 copies (*n*, %)	no SMA	7 (4.6%)	3 (5.4%)	4 (4.1%)	402 (100%)
	2	46 (30.1%)	42 (75.0%)	4 (4.1%)	-
	3	68 (44.4%)	10 (17.9%)	58 (59.8%)	-
	4	32 (20.9%)	1 (1.8%)	31 (32.0%)	-
Motorscores [points] (*n*, mean ± SD)	unreported	72 (47.1%)	22 (39.2%)	50 (51.5%)	402 (100%)
	CHOP-INTEND	32.21 (±11.70) (*n* = 39)	31.88 (±12.00) (*n* = 32)	33.71 (±10.92) ^1^ (*n* = 7)	-
	HFMSE	25.00 (±20.11) (*n* = 42)	8.50 (±2.12) (*n* = 2)	25.83 (±20.26) (*n* = 40)	-

^1^ CHOP-INTEND was reported in 7/97 patients in G12.1 group. Mean age was 6.32 (±5.34) years; all patients were reported as having SMA type II.

## Data Availability

Biomaterials are available upon request to the corresponding author for other research projects. To protect patient data in our vulnerable group, clinical and spectroscopy data are only available upon justified request to the corresponding author.

## Supplementary Materials

The following supporting information can be downloaded at https://www.mdpi.com/article/10.3390/ijms252212123/s1.

## References

[1] Wirth B. (2021). Spinal Muscular Atrophy: In the Challenge Lies a Solution. Trends Neurosci..

[2] Lunn M.R., Wang C.H. (2008). Spinal Muscular Atrophy. Lancet Seminar Spinal Muscular Atrophy. Lancet.

[3] Mercuri E., Finkel R.S., Muntoni F., Wirth B., Montes J., Main M., Mazzone E.S., Vitale M., Snyder B., Quijano-Roy S. (2018). Diagnosis and Management of Spinal Muscular Atrophy: Part 1: Recommendations for Diagnosis, Rehabilitation, Orthopedic and Nutritional Care. Neuromuscul. Disord..

[4] Erdos J., Wild C. (2022). Mid- and Long-Term (at Least 12 Months) Follow-up of Patients with Spinal Muscular Atrophy (SMA) Treated with Nusinersen, Onasemnogene Abeparvovec, Risdiplam or Combination Therapies: A Systematic Review of Real-World Study Data. Eur. J. Paediatr. Neurol..

[5] Pechmann A., Behrens M., Dörnbrack K., Tassoni A., Wenzel F., Stein S., Vogt S., Zöller D., Bernert G., Hagenacker T. (2022). Improved Upper Limb Function in Non-Ambulant Children with SMA Type 2 and 3 during Nusinersen Treatment: A Prospective 3-Years SMArtCARE Registry Study. Orphanet J. Rare Dis..

[6] Farrar M.A., Teoh H.L., Carey K.A., Cairns A., Forbes R., Herbert K., Holland S., Jones K.J., Menezes M.P., Morrison M. (2018). Nusinersen for SMA: Expanded Access Programme. J. Neurol. Neurosurg. Psychiatry.

[7] Pane M., Palermo C., Messina S., Sansone V.A., Bruno C., Catteruccia M., Sframeli M., Albamonte E., Pedemonte M., D’Amico A. (2018). Nusinersen in Type 1 SMA Infants, Children and Young Adults: Preliminary Results on Motor Function. Neuromuscul. Disord..

[8] Pechmann A., Langer T., Schorling D., Stein S., Vogt S., Schara U., Kolbel H., Schwartz O., Hahn A., Giese K. (2018). Evaluation of Children with SMA Type 1 Under Treatment with Nusinersen within the Expanded Access Program in Germany. J. Neuromuscul. Dis..

[9] Strauss K.A., Farrar M.A., Muntoni F., Saito K., Mendell J.R., Servais L., McMillan H.J., Finkel R.S., Swoboda K.J., Kwon J.M. (2022). Onasemnogene Abeparvovec for Presymptomatic Infants with Three Copies of SMN2 at Risk for Spinal Muscular Atrophy: The Phase III SPR1NT Trial. Nat. Med..

[10] Vill K., Kolbel H., Schwartz O., Blaschek A., Olgemoller B., Harms E., Burggraf S., Roschinger W., Durner J., Glaser D. (2019). One Year of Newborn Screening for SMA—Results of a German Pilot Project. J. Neuromuscul. Dis..

[11] Vill K., Schwartz O., Blaschek A., Gläser D., Nennstiel U., Wirth B., Burggraf S., Röschinger W., Becker M., Czibere L. (2021). Newborn Screening for Spinal Muscular Atrophy in Germany: Clinical Results after 2 Years. Orphanet J. Rare Dis..

[12] Schwartz O., Vill K., Pfaffenlehner M., Behrens M., Weiß C., Johannsen J., Friese J., Hahn A., Ziegler A., Illsinger S. (2024). Clinical Effectiveness of Newborn Screening for Spinal Muscular Atrophy: A Nonrandomized Controlled Trial. JAMA Pediatr..

[13] Blaschek A., Kölbel H., Schwartz O., Köhler C., Gläser D., Eggermann K., Hannibal I., Schara-Schmidt U., Müller-Felber W., Vill K. (2022). Newborn Screening for SMA—Can a Wait-and-See Strategy Be Responsibly Justified in Patients with Four SMN2 Copies?. J. Neuromuscul. Dis..

[14] Bromberg M.B., Swoboda K.J., Lawson V.H. (2003). Counting Motor Units in Chronic Motor Neuropathies. Exp. Neurol..

[15] Galea V., Fehlings D., Kirsch S., McComas A. (2001). Depletion and Sizes of Motor Units in Spinal Muscular Atrophy. Muscle Nerve.

[16] Kolb S.J., Coffey C.S., Yankey J.W., Krosschell K., Arnold W.D., Rutkove S.B., Swoboda K.J., Reyna S.P., Sakonju A., Darras B.T. (2017). Natural History of Infantile-Onset Spinal Muscular Atrophy. Ann. Neurol..

[17] Simard L.R., Belanger M.C., Morissette S., Wride M., Prior T.W., Swoboda K.J. (2007). Preclinical Validation of a Multiplex Real-Time Assay to Quantify SMN MRNA in Patients with SMA. Neurology.

[18] Sumner C.J., Kolb S.J., Harmison G.G., Jeffries N.O., Schadt K., Finkel R.S., Dreyfuss G., Fischbeck K.H. (2006). SMN MRNA and Protein Levels in Peripheral Blood: Biomarkers for SMA Clinical Trials. Neurology.

[19] Chabanon A., Seferian A.M., Daron A., Pereon Y., Cances C., Vuillerot C., De Waele L., Cuisset J.M., Laugel V., Schara U. (2018). Prospective and Longitudinal Natural History Study of Patients with Type 2 and 3 Spinal Muscular Atrophy: Baseline Data NatHis-SMA Study. PLoS ONE.

[20] Alves C.R.R., Zhang R., Johnstone A.J., Garner R., Nwe P.H., Siranosian J.J., Swoboda K.J. (2020). Serum Creatinine Is a Biomarker of Progressive Denervation in Spinal Muscular Atrophy. Neurology.

[21] Alves C.R.R., Zhang R., Johnstone A.J., Garner R., Eichelberger E.J., Lepez S., Yi V., Stevens V., Poxson R., Schwartz R. (2020). Whole Blood Survival Motor Neuron Protein Levels Correlate with Severity of Denervation in Spinal Muscular Atrophy. Muscle Nerve.

[22] Darras B.T., Crawford T.O., Finkel R.S., Mercuri E., De Vivo D.C., Oskoui M., Tizzano E.F., Ryan M.M., Muntoni F., Zhao G. (2019). Neurofilament as a Potential Biomarker for Spinal Muscular Atrophy. Ann. Clin. Transl. Neurol..

[23] Saffari A., Cannet C., Blaschek A., Hahn A., Hoffmann G.F., Johannsen J., Kirsten R., Kockaya M., Kölker S., Müller-Felber W. (2021). 1H-NMR-Based Metabolic Profiling Identifies Non-Invasive Diagnostic and Predictive Urinary Fingerprints in 5q Spinal Muscular Atrophy. Orphanet J. Rare Dis..

[24] Glascock J., Sampson J., Connolly A.M., Darras B.T., Day J.W., Finkel R., Howell R.R., Klinger K.W., Kuntz N., Prior T. (2020). Revised Recommendations for the Treatment of Infants Diagnosed with Spinal Muscular Atrophy Via Newborn Screening Who Have 4 Copies of SMN2. J. Neuromuscul. Dis..

[25] Abiusia E., Costa-Rogerb M., Bertinic E.S., Tizianoa F.D., Tizzanob E.F., Abiusi E., Baranello G., Bertini E., Boemer F., SMN2 Study Group (2024). 270th ENMC International Workshop: Consensus for SMN2 Genetic Analysis in SMA Patients. Neuromuscul. Disord..

[26] Dosi C., Masson R. (2024). The Impact of Three SMN2 Gene Copies on Clinical Characteristics and Effect of Disease-Modifying Treatment in Patients with Spinal Muscular Atrophy: A Systematic Literature Review. Front. Neurol..

[27] Weng W.-C., Hsu Y.-K., Chang F.-M., Lin C.-Y., Hwu W.-L., Lee W.-T., Lee N.-C., Chien Y.-H. (2021). CMAP Changes upon Symptom Onset and during Treatment in Spinal Muscular Atrophy Patients: Lessons Learned from Newborn Screening. Genet. Med..

[28] Lori S., Bertini G., Gabbanini S., Bastianelli M., Cossu C., Lolli F., Dani C. (2020). Neuromuscular Maturation in the Neonate: Combined Electroneurographic and Ultrasonographic Study. Early Hum. Dev..

[29] Marshall D.D., Powers R. (2017). Beyond the Paradigm: Combining Mass Spectrometry and Nuclear Magnetic Resonance for Metabolomics. Prog. Nucl. Magn. Reson. Spectrosc..

[30] Larive C.K., Barding G.A., Dinges M.M. (2015). NMR Spectroscopy for Metabolomics and Metabolic Profiling. Anal. Chem..

[31] Wishart D.S., Cheng L.L., Copié V., Edison A.S., Eghbalnia H.R., Hoch J.C., Gouveia G.J., Pathmasiri W., Powers R., Schock T.B. (2022). NMR and Metabolomics—A Roadmap for the Future. Metabolites.

[32] Lu Y., Pang Z., Xia J. (2023). Comprehensive Investigation of Pathway Enrichment Methods for Functional Interpretation of LC-MS Global Metabolomics Data. Brief. Bioinform..

[33] Uhlén M., Fagerberg L., Hallström B.M., Lindskog C., Oksvold P., Mardinoglu A., Sivertsson Å., Kampf C., Sjöstedt E., Asplund A. (2015). Tissue-Based Map of the Human Proteome. Science.

[34] Tapken I., Schweitzer T., Paganin M., Schüning T., Detering N.T., Sharma G., Niesert M., Saffari A., Kuhn D., Glynn A. (2024). The Systemic Complexity of a Monogenic Disease: The Molecular Network of Spinal Muscular Atrophy. Brain.

[35] Bowerman M., Swoboda K.J., Michalski J.-P., Wang G.-S., Reeks C., Beauvais A., Murphy K., Woulfe J., Screaton R.A., Scott F.W. (2012). Glucose Metabolism and Pancreatic Defects in Spinal Muscular Atrophy. Ann. Neurol..

[36] Djordjevic S.A., Milic-Rasic V., Brankovic V., Kosac A., Dejanovic-Djordjevic I., Markovic-Denic L., Djuricic G., Milcanovic N., Kovacevic S., Petrovic H. (2021). Glucose and Lipid Metabolism Disorders in Children and Adolescents with Spinal Muscular Atrophy Types 2 and 3. Neuromuscul. Disord..

[37] Errico F., Marino C., Grimaldi M., Nuzzo T., Bassareo V., Valsecchi V., Panicucci C., Di Schiavi E., Mazza T., Bruno C. (2022). Nusinersen Induces Disease-Severity-Specific Neurometabolic Effects in Spinal Muscular Atrophy. Biomolecules.

[38] Wu J., Gao Y. (2015). Physiological Conditions Can Be Reflected in Human Urine Proteome and Metabolome. Expert Rev. Proteom..

[39] Deutsch L., Osredkar D., Plavec J., Stres B. (2021). Spinal Muscular Atrophy after Nusinersen Therapy: Improved Physiology in Pediatric Patients with No Significant Change in Urine, Serum, and Liquor 1H-NMR Metabolomes in Comparison to an Age-Matched, Healthy Cohort. Metabolites.

[40] Federal Institute for Drugs and Medical Devices (BfArM), on behalf of the Federal Ministry of Health (BMG), ICD Working Group of the Board of Trustees for Questions of Classification in Health Care (KKG) (2022). ICD-10-GM: International Statistical Classification of Diseases and Related Health Problems.

[41] (2016). Molecular In Vitro Diagnostic Examinations—Specivifaction for Pre-examination Processes for Metabolomics in Urine, Venous Blood Serum and Plasma.

[42] Dona A.C., Jiménez B., Schafer H., Humpfer E., Spraul M., Lewis M.R., Pearce J.T.M., Holmes E., Lindon J.C., Nicholson J.K. (2014). Precision High-Throughput Proton NMR Spectroscopy of Human Urine, Serum, and Plasma for Large-Scale Metabolic Phenotyping. Anal. Chem..

[43] Berezhnoy G., Laske C., Trautwein C. (2023). Metabolomic Profiling of CSF and Blood Serum Elucidates General and Sex-Specific Patterns for Mild Cognitive Impairment and Alzheimer’s Disease Patients. Front. Aging Neurosci..

[44] Wider G., Dreier L. (2006). Measuring Protein Concentrations by NMR Spectroscopy. J. Am. Chem. Soc..

[45] Assfalg M., Bertini I., Colangiuli D., Luchinat C., Schäfer H., Schütz B., Spraul M. (2008). Evidence of Different Metabolic Phenotypes in Humans. Proc. Natl. Acad. Sci. USA.

[46] Bernini P., Bertini I., Luchinat C., Nepi S., Saccenti E., Schäfer H., Schütz B., Spraul M., Tenori L. (2009). Individual Human Phenotypes in Metabolic Space and Time. J. Proteome Res..

[47] Xia J., Wishart D.S. (2011). Metabolomic Data Processing, Analysis, and Interpretation Using MetaboAnalyst. Curr. Protoc. Bioinform..

[48] Lin G., Dong L., Cheng K.K., Xu X., Wang Y., Deng L., Raftery D., Dong J. (2023). Differential Correlations Informed Metabolite Set Enrichment Analysis to Decipher Metabolic Heterogeneity of Disease. Anal. Chem..

[49] Goeman J.J., van de Geer S.A., de Kort F., van Houwelingen H.C. (2004). A Global Test for Groups of Genes: Testing Association with a Clinical Outcome. Bioinformatics.

