# Supplementary material for: Identification of Biochemical Determinants for Diagnosis and Prediction of Severity in 5q Spinal Muscular Atrophy Using 1H-Nuclear Magnetic Resonance Metabolic Profiling in Patient-Derived Biofluids
Source: Int J Mol Sci. 2024 Nov 12;25(22):12123. doi: 10.3390/ijms252212123 (PMC11594255; doi:10.3390/ijms252212123)
Supplement: Supplementary file 1 [file ijms-25-12123-s001.zip › ijms-3266048-supplementary.pdf]

SUPPLEMENTARY MATERIAL

Figure S1: Age distribution among different biomaterials

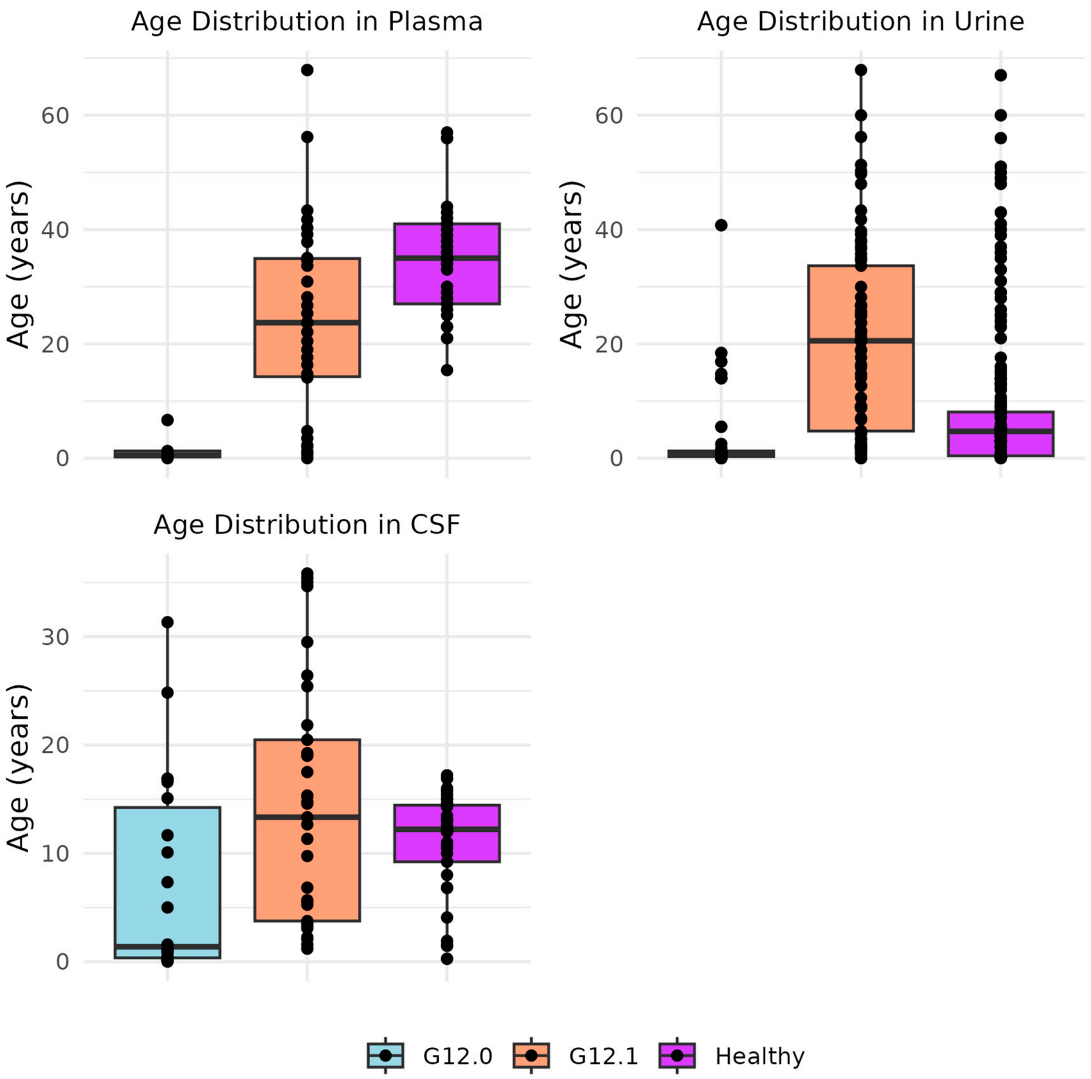

Table S1 Recruited patients and contributed biomaterial by each center

	**Plasma**		**Urine**		**CSF**		**Individual patients**	
	G12.0	G12.1	G12.0	G12.1	G12.0	G12.1	G12.0	G12.1
Essen	0	0	1	6	0	0	1	6
Giessen	0	0	0	0	18	24	18	23
Hamburg	1	1	3	4	0	0	3	4
Heidelberg	7	29	33	56	7	10	32	53
Munich	0	0	2	11	0	0	2	11

The study and was approved by the ethics committees of the Universities of Essen (19-9011-BO; date 30.04.2020), Giessen (AZ 166/20; date 30.09.2020), Heidelberg (S-554/2018; date 26.10.2018), Hamburg (MC-265/19: date 10.09.2019) and Munich (18-269; date 19.6.2019) and the ethics committee of the State Medical Council of Baden Württemberg (F-2013-006#A, date 10.01.2020). All patients and their legal guardians enrolled in this study provided signed informed consent. All healthy control samples were collected at the Heidelberg partner site.

Table S2 A-C. : Results of the quantitative enrichment analysis G12.0 vs. G12.1

(A) Urine

	**Compounds**	**Hits**	**StatisticQ**	**ExpectedQ**	**Raw p**	**Holm corrected p**	**FDR**
Citrate cycle (TCA cycle)	7	7	10,40	0,93	0,000	0,000	0,000
Alanine, aspartate and glutamate metabolism	10	9	8,80	0,93	0,000	0,000	0,000
Starch and sucrose metabolism	1	1	17,88	0,93	0,000	0,000	0,000
Neomycin, kanamycin and gentamicin biosynthesis	1	1	17,88	0,93	0,000	0,000	0,000
Galactose metabolism	6	4	8,42	0,93	0,000	0,000	0,000
Glyoxylate and dicarboxylate metabolism	8	8	8,04	0,93	0,000	0,000	0,000
Primary bile acid biosynthesis	2	2	11,44	0,93	0,000	0,001	0,000
Taurine and hypotaurine metabolism	1	1	15,00	0,93	0,000	0,001	0,000
Purine metabolism	5	4	6,03	0,93	0,000	0,001	0,000
Nicotinate and nicotinamide metabolism	2	1	14,21	0,93	0,000	0,001	0,000

Glycine, serine and threonine metabolism	10	9	6,62	0,93	0,000	0,002	0,000
Tyrosine metabolism	7	4	6,05	0,93	0,000	0,003	0,000
Pyruvate metabolism	5	5	6,06	0,93	0,000	0,003	0,000
Lipoic acid metabolism	3	3	9,05	0,93	0,000	0,003	0,000
Arginine biosynthesis	7	4	5,93	0,93	0,000	0,005	0,001
Pantothenate and CoA biosynthesis	5	3	6,74	0,93	0,000	0,008	0,001
Arginine and proline metabolism	5	4	5,55	0,93	0,001	0,015	0,002
Caffeine metabolism	2	2	8,23	0,93	0,001	0,021	0,002
Valine, leucine and isoleucine biosynthesis	7	5	4,38	0,93	0,002	0,036	0,004
Propanoate metabolism	4	3	4,39	0,93	0,002	0,036	0,004
Cysteine and methionine metabolism	3	2	5,64	0,93	0,002	0,036	0,004
Glycolysis / Gluconeogenesis	3	3	5,40	0,93	0,002	0,036	0,004
Butanoate metabolism	6	6	3,65	0,93	0,003	0,042	0,004
Valine, leucine and isoleucine degradation	8	7	3,19	0,93	0,005	0,073	0,008
Glutathione metabolism	2	2	4,47	0,93	0,007	0,093	0,011
Porphyrin metabolism	2	2	4,39	0,93	0,008	0,093	0,011
D-Amino acid metabolism	1	1	5,37	0,93	0,015	0,168	0,021
Fructose and mannose metabolism	2	2	1,83	0,93	0,138	1,000	0,177
Amino sugar and nucleotide sugar metabolism	2	2	1,83	0,93	0,138	1,000	0,177
Pentose phosphate pathway	1	1	1,98	0,93	0,144	1,000	0,178
Pyrimidine metabolism	6	5	1,48	0,93	0,156	1,000	0,187
Phenylalanine, tyrosine and tryptophan biosynthesis	4	2	1,68	0,93	0,170	1,000	0,197
Vitamin B6 metabolism	1	1	1,67	0,93	0,181	1,000	0,203
beta-Alanine metabolism	4	2	1,41	0,93	0,214	1,000	0,233
Phenylalanine metabolism	5	4	1,32	0,93	0,223	1,000	0,236
Nitrogen metabolism	1	1	0,01	0,93	0,930	1,000	0,956
Glycerophospholipid metabolism	1	1	0,00	0,93	0,970	1,000	0,970

(B) Plasma

	Compounds	Hits	StatisticQ	ExpectedQ	Raw p	Holm corrected p	FDR
Glycerolipid metabolism	1	1	19,78	2,86	0,007	0,223	0,111
Glyoxylate and dicarboxylate metabolism	6	6	7,45	2,86	0,014	0,456	0,111
Histidine metabolism	1	1	15,47	2,86	0,018	0,564	0,111
beta-Alanine metabolism	1	1	15,47	2,86	0,018	0,564	0,111
Valine, leucine and isoleucine biosynthesis	4	4	10,15	2,86	0,020	0,587	0,111
Galactose metabolism	3	2	10,76	2,86	0,020	0,587	0,111
Glycine, serine and threonine metabolism	7	7	6,35	2,86	0,046	1,000	0,213
Tyrosine metabolism	3	3	7,48	2,86	0,050	1,000	0,213
Butanoate metabolism	4	4	7,28	2,86	0,060	1,000	0,226
Valine, leucine and isoleucine degradation	4	4	7,02	2,86	0,066	1,000	0,226
Propanoate metabolism	1	1	7,52	2,86	0,106	1,000	0,326
Ubiquinone and other terpenoid-quinone biosynthesis	1	1	6,64	2,86	0,129	1,000	0,366
Citrate cycle (TCA cycle)	4	4	4,54	2,86	0,174	1,000	0,425
Cysteine and methionine metabolism	2	2	4,46	2,86	0,213	1,000	0,425
Phenylalanine metabolism	2	2	4,41	2,86	0,213	1,000	0,425
Phenylalanine, tyrosine and tryptophan biosynthesis	2	2	4,41	2,86	0,213	1,000	0,425
Lysine degradation	1	1	4,29	2,86	0,225	1,000	0,425
Biotin metabolism	1	1	4,29	2,86	0,225	1,000	0,425
Glycolysis / Gluconeogenesis	3	3	3,75	2,86	0,270	1,000	0,459
Pyruvate metabolism	3	3	3,75	2,86	0,270	1,000	0,459
Lipoic acid metabolism	3	3	3,53	2,86	0,299	1,000	0,485
Alanine, aspartate and glutamate metabolism	6	6	3,37	2,86	0,318	1,000	0,491
Starch and sucrose metabolism	1	1	1,75	2,86	0,442	1,000	0,580
Neomycin, kanamycin and gentamicin biosynthesis	1	1	1,75	2,86	0,442	1,000	0,580
Arginine and proline metabolism	4	4	2,58	2,86	0,471	1,000	0,580
Purine metabolism	1	1	1,49	2,86	0,478	1,000	0,580
Pyrimidine metabolism	1	1	1,49	2,86	0,478	1,000	0,580
Nitrogen metabolism	1	1	1,49	2,86	0,478	1,000	0,580
Glycerophospholipid metabolism	1	1	1,34	2,86	0,502	1,000	0,588
Arginine biosynthesis	3	3	1,68	2,86	0,639	1,000	0,724
Pantothenate and CoA biosynthesis	1	1	0,16	2,86	0,818	1,000	0,897
Primary bile acid biosynthesis	1	1	0,05	2,86	0,896	1,000	0,904
Porphyrin metabolism	1	1	0,05	2,86	0,896	1,000	0,904
Glutathione metabolism	2	2	0,29	2,86	0,904	1,000	0,904

Phenylalanine metabolism	5	4	1,32	0,93	0,223	1,000	0,236
Nitrogen metabolism	1	1	0,01	0,93	0,930	1,000	0,956
Glycerophospholipid metabolism	1	1	0,00	0,93	0,970	1,000	0,970

(C) CSF

	**Compounds**	**Hits**	**StatisticQ**	**ExpectedQ**	**Raw p**	**Holm corrected p**	**FDR**
Ascorbate and aldarate metabolism	**1**	**1**	**10,44**	**1,75**	**0,013**	**0,375**	**0,187**
Inositol phosphate metabolism	1	1	10,44	1,75	0,013	0,375	0,187
Galactose metabolism	4	4	3,09	1,75	0,136	1,000	0,910
Phenylalanine metabolism	1	1	1,73	1,75	0,325	1,000	0,910
Phenylalanine, tyrosine and tryptophan biosynthesis	1	1	1,73	1,75	0,325	1,000	0,910
Primary bile acid biosynthesis	1	1	1,72	1,75	0,327	1,000	0,910
Glutathione metabolism	1	1	1,72	1,75	0,327	1,000	0,910
Porphyrin metabolism	1	1	1,72	1,75	0,327	1,000	0,910
Cysteine and methionine metabolism	2	2	1,76	1,75	0,374	1,000	0,910
Glycerolipid metabolism	1	1	1,13	1,75	0,427	1,000	0,910
Starch and sucrose metabolism	1	1	0,76	1,75	0,516	1,000	0,910
Neomycin, kanamycin and gentamicin biosynthesis	1	1	0,76	1,75	0,516	1,000	0,910
Butanoate metabolism	2	2	0,89	1,75	0,576	1,000	0,910
Lipoic acid metabolism	2	2	0,87	1,75	0,612	1,000	0,910
Valine, leucine and isoleucine biosynthesis	3	3	0,97	1,75	0,646	1,000	0,910
Pyruvate metabolism	3	3	0,91	1,75	0,678	1,000	0,910
Glycolysis / Gluconeogenesis	3	3	0,91	1,75	0,678	1,000	0,910
Arginine and proline metabolism	2	2	0,55	1,75	0,739	1,000	0,910
Pantothenate and CoA biosynthesis	2	2	0,47	1,75	0,769	1,000	0,910
Valine, leucine and isoleucine degradation	4	4	0,74	1,75	0,791	1,000	0,910
Glyoxylate and dicarboxylate metabolism	6	5	0,63	1,75	0,796	1,000	0,910
Glycine, serine and threonine metabolism	4	4	0,71	1,75	0,798	1,000	0,910
Citrate cycle (TCA cycle)	2	2	0,27	1,75	0,861	1,000	0,910
Alanine, aspartate and glutamate metabolism	3	2	0,27	1,75	0,861	1,000	0,910
Fructose and mannose metabolism	1	1	0,05	1,75	0,870	1,000	0,910
Amino sugar and nucleotide sugar metabolism	1	1	0,05	1,75	0,870	1,000	0,910
Glycerophospholipid metabolism	1	1	0,04	1,75	0,878	1,000	0,910
Tyrosine metabolism	2	2	0,04	1,75	0,978	1,000	0,978
